# Nursing students’ approaches to learning in selected Malawian nursing schools: a cross-sectional study

**DOI:** 10.1186/s12909-024-05746-y

**Published:** 2024-07-12

**Authors:** Dalitso Zolowere Chitokoto, Noel Dzinnenani Mbirimtengerenji, Lucy Ida Kululanga

**Affiliations:** 1 Ministry of Health, Rumphi District Hospital, P.O Box 225, Rumphi, Malawi; 2 School of Nursing, Kamuzu University of Health Sciences (KUHeS), Lilongwe, Malawi

**Keywords:** Approaches to learning, Deep learning, Surface learning, Motive, Strategy

## Abstract

**Background:**

Students’ approaches to learning are of essence in nursing education. This is because nursing is a profession where classroom learning leads to clinical performance. Although the literature recognizes student’s approaches to learning as a significant aspect affecting the quality of students’ learning, studies suggest that quality of learning has not been highly achieved in Malawian nursing colleges. Currently, there is a scarcity of empirical data on the learning approaches that Malawian nursing and midwifery students in nursing colleges employ. This study assessed the different approaches to learning among nursing and midwifery students in selected Malawian nursing colleges.

**Methods:**

This was a cross- sectional study that employed quantitative methods. The target population was nursing and midwifery students pursuing nursing diplomas from Nkhoma College of Nursing, Ekwendeni College of Health Sciences and Malawi College of Health Sciences. A total of 251 students were sampled randomly from the three nursing colleges. Data was collected through a self-administered questionnaire (R-SPQ-2 F) by Biggs. The data was analyzed using chi-square and binary logistic regression. In this study Cronbach’s alpha was 0.6.

**Results:**

Most students had used a deep approach to learning (M = 3.201, SD = 0.623) than the surface approach (M = 2.757, SD = 0.732). Being in the age category of 16–20 had more likelihood of adopting a surface approach to learning compared to other age categories (X^2^ = 7.669, DF 2, *P* = .02). Students from Malawi College of Health Sciences were more likely to adopt a surface approach to learning compared to students from Nkhoma Nursing College and Ekwendeni College of Health Sciences (X^2^ = 12.388, *df* = 2, *P* = .002).

**Conclusion:**

A deep approach to learning emerged as the most preferred approach to learning which indirectly implies that most students attain meaningful learning. Age and environment are some of the key determinants associated with different learning approaches. More attention should be given to younger students during teaching and learning to promote deep learning.

## Background

Quality learning outcomes in nursing and midwifery education are reflected in nurses and midwives’ ability to think critically and professionally manage patients in diverse health care settings. It is evident that approaches to learning are a significant aspect affecting the quality of a student’s learning, [1, 2, 3]. Student approaches to learning were originally defined as distinct ways in which students face their academic tasks [4]. There are two student approaches to learning being focused on in this study namely deep and surface. A deep approach to learning is an approach in which students involve themselves meaningfully with the subject matter intending to attain a meaningful understanding of the content through reading, relating prior knowledge and personal experiences to the learnt content [5]. A surface approach to learning, on the other hand, is one in which a student learns just enough to pass an assessment with the intention to fulfill the minimum requirements of an educational programme without an in-depth understanding of the learnt content, usually relying on memorizing and reproduction of learnt content [6, 7]. There is also a strategic approach to learning and students with a strategic approach to learning, are focused on achievement on assessments, and as such pay much attention to the organizing and management of their study efforts so that they achieve better grades [8]. Students with a strategic approach use both deep and surface approach to learning and focus on achieving good grades. This approach to learning was not used in the analysis of this study results because this study is using the Revised Two- Factor Study Process Questionnaire which has only the two approaches. It should however be noted that an approach to learning shows the relationship between the learner, the context and the learning task [9]. This means the student’s demographic characteristics like age and the approach that nurse educators use when teaching in terms of teaching strategies and assessment affects the student’s adoption of a learning approach.

Students use both surface and deep approaches to learning alike and are able to capitalize on either learning approach and there are significant difference between students’ age, gender and their adopted approach to learning with older age and being female having higher scores for a deep approach to learning than younger and male students [10, 11]. This means students have the ability of using either of the approaches to learning. Deep learning approaches significantly predict good academic performance, while surface learning approaches significantly predict poor academic performance [12, 13, 14]. Furthermore, Malcom Knowles’s principles of adult learning state that adults are self-motivated, self-directed, ready to learn, and have experience that they relate to what they are learning [15, 16]. This could put older students at a higher chance of having a deep approach to learning than younger students who have not transitioned to adulthood. On the contrast a study conducted in Singapore, which aimed to assess the predominant learning approaches of medical students found that there was no significant difference between students’ age, sex, and level of qualification/year of study and adopting either the deep or surface approach [17]. This means that how students adopt an approach to learning varies from one college to another.

The teaching approach also affects the students’ approaches to learning [18]. Teaching approaches are described in terms of student-centered and teacher-centered approaches. Student-centered teaching approaches promote a student-centered learning environment, which in turn promotes deep approaches to learning, whereas a teacher-centered learning environment promotes surface approaches to learning [19]. In this regard, it is important for teachers to teach using student-centered approaches. A study was conducted in Botswana, Ghana, and Kenya and concluded that an approach that a student adopts affects his/her ability to think critically [20]. They further explained that students who adopted a deep approach to learning had higher critical thinking scores, whereas those adopting a surface approach to learning had lower critical thinking scores. Thus, it is imperative for nursing and midwifery educators to be mindful of what approaches to learning the students they teach adopt [21]. The difference in how students approach learning is significant in nursing education as it affects learning outcomes and has a great impact on nursing care and patient outcomes. Therefore, there is need to be aware of the various approaches available in a group of students. Previous findings show that most students adopt a deep approach to learning as they advance with their education [22, 23]. However, it was also discovered that it is not certain that students in higher education develop towards the deep approach. Students in higher education can either develop towards deep or surface approaches.

A study conducted in Malawi on Malawian nursing students, revealed that both deep and surface approaches to learning were prevalent among Malawian Bachelor of Science in nursing students with deep learning approach being the most prevalent in about 79% of the students [24]. However, a study by Mbirimtengerenji and Adejumo which focused on utilization of teaching strategies among nurse tutors in Malawian nursing colleges discovered that there is compromised quality of learning among students in the colleges [25]. Effective use of teaching strategies affects the students’ approaches to learning.

Previous studies have revealed that student approaches to learning is one aspect that affects the quality of learning outcomes and that students’ approaches to learning can change depending on the context [5, 11, 22]. This means it is of paramount importance to always check the approaches to learning that students adopt while learning. This stimulated the researchers to study the nursing students’ approaches to learning in Malawi. In addition, there was still scarcity of data on what approaches to learning were prevalent among Malawian nursing and midwifery students which this study was trying to uncover. The study findings have generated new knowledge in the area of student nurses’ approaches to learning in the Malawian context. It might set a basis for further research on student nurses’ approaches to learning. Having students who adopt a deep approach to learning would also improve service delivery when they qualify as these students will be self-directed, life-long learners who can tackle the challenges of dynamism in health care. In addition, the findings of this study might provide a basis for policy development in selected nursing colleges.

## Methods

### Study design

A quantitative descriptive cross -sectional study was conducted between June 2019 and August, 2021.

### Study population

The target population was diploma nursing and midwifery students in year one, two and three from Nkhoma College of Nursing, Ekwendeni College of Health Sciences and Malawi College of Health Sciences.

### Sampling

The three nursing colleges were randomly selected from the 10 nursing schools that offer nursing and midwifery education in Malawi. From each selected college (cluster) three strata were chosen, namely: year one, year two and year three. Using class registers from each year (class), students’ names were picked at random until the sample from each stratum in each cluster was reached. Sample size was calculated using the Slovin’s formula. A total of 251 students were sampled from the three nursing colleges using simple random selection.

### Data collection instrument

Data was collected through a self-administered questionnaire (R-SPQ-2 F) that was developed by John Biggs containing 20 items representing two main scales of learning approaches, deep and surface, with four subscales: deep motive, deep strategy, surface motive, and surface strategy. Each subscale had five items and each item was rated on a 5-point Likert scale [26]. The responses total scores were found using scoring system provided by Biggs [7], which says:

- Deep approach score: Σ all deep motive scores + all deep strategy scores.
- Surface approach score: Σ All surface motive scores + all surface strategy scores.

The total scores for each sub-category add up to 25 marks, the deep approach and surface approach total scores add up to 50 each and the whole questionnaire yields 100 marks.

### Validation of data collection instrument

Pre-testing was done at St Johns Institute for Health Sciences which was not among the three selected colleges but had students pursuing the same nursing and midwifery diploma as the study participants. Thirty nursing and midwifery students were sampled and given the questionnaires. The students were from year one, two and three. All the questions were completed and the questionnaire was found to be relevant to the research at hand as such no question was changed from the questionnaire. In this study population, R-SPQ-2 F had overall Cronbach’s alpha value of 0.60 for its 20 items. The deep and surface approach total scales were found to have 0.60 and 0.68, respectively and the R-SPQ-2 F motive and strategy subscales. The original questionnaire had a Cronbach’s alpha value of 0.70 for the whole questionnaire thus a total Cronbach’s alpha found in this study 0.60. A Cronbach’s alpha of 6.0 and above is acceptable [27, 28].

### Data collection and management

Data was collected from 2nd to 28th February, 2021. The first author collected data with assistance of a research assistant who had prior experience in students’ learning research. The research assistant had to undergo a training before starting data collection to ensure consistency in data collection and adherence to ethical considerations.

Participants were found in their respective colleges. Recruitment of the participants was done with the assistance of the respective college principals and it was on voluntary basis. The students were verbally briefed on the study, its significance as well as benefits of participating in the study. The class registers were used to select students’ numbers at random to participate in the study. The sampled students were given the participant information sheet, consent form and encouraged to ask questions where they needed clarification. Students that were willing to participate were asked to sign the consent form. Since data collection was done during the COVID-19 pandemic, all preventive measures were followed such as wearing of masks, hand hygiene, and social distancing.

Questionnaires with responses were kept in a lockable cabinet with a lock which its keys were kept by the principal investigator only and the data was put in a folder in the principal investigator’s laptop in a folder that had a password known by the principal investigator only in order to prevent others from accessing the data and altering it. A duplicate folder was kept in the principal investigator’s flash disk which also had a password only known to her for backup. Data were coded, entered into Statistical Package for Social Sciences (SPSS) version 26.0. Data cleaning was done by checking the missing values and outliers and when found reference was made to the questionnaire in question and correct recording was done.

### Data analysis

Descriptive statistics such as frequencies, percentages and means, were used to calculate demographic data and approaches to learning variables using the above explained scoring methods. Chi square test was run to see if there was association between students’ demographic characteristics and their approaches to learning. Binary logistic regression was done to identify factors that influenced the different approaches to learning.

## Results

### Demographics results

A total of 251 questionnaires were distributed and all of them were completed representing a 100% response rate. Among the respondents, the majority were females (55.4%) which was consistent with the composition of the study population. The respondents were from Ekwendeni College of Health Sciences (31.5%), Malawi College of Health Sciences (Zomba campus) (30.3%) and Nkhoma College of Nursing (38.2%). They were within the 16–20 (15.9%), 21–30 (77.7%) and 31–40 (6.4%) years’ age groups and in first (39%), second (34.7%) and third (26.3%) years of study.

### Students’ approaches to learning among the participants

A larger population of the students had used a deep approach to learning which was shown by the mean for deep approach being higher than that of surface approach to learning. There was a statistically significant difference in the students’ adoption of the deep approach (M = 3.201, SD = 0.623) and the surface approach (M = 2.757, SD = 0.732). The 95% confidence interval between the means ranged from = -0.569- -0.568 and this indicated a difference between the mean of surface and deep approaches to learning. This means the students differed in how they adopted the different approaches to learning with most students adopting the deep approach to learning (See Table 1).

**Table 1:** Results of student approaches to learning

Scales	Mean	SD
Deep approach	3.201	0.623
Surface approach	2.757	0.732

### Association of students’ demographic characteristics and their adopted approaches to learning

There was statistical significance between age of student, college of study and surface approach to learning (*df* = 2, *N* = 251, X^2^ = 7.669, *P* = .02 and X^2^ = *df* = 2, *N* = 251, X^2^ = 12.388, *P* = .002 respectively) (See Table 2). Students within the age 21–30 years category were less likely to adopt a surface approach to learning compared those within 16–20 years age category and there was statistical significance (OR = 0.253, 95% CI = 0.11–0.582 and *P* = .001). This means students within the 16–20 years group were more likely to adopt the surface approach more the other age categories. Students within the age 31years and above category were also less likely to adopt the surface approach to learning compared to students within the age 16- 20years category but there was no statistical significance (OR = 0.463, 95% CI = 0.121-1.765 and *P* = .259). Nkhoma College of nursing students were more likely to adopt a surface approach to learning than students from Ekwendeni College of Health Sciences, however there was no statistical significance (OR = 1.448, 95% CI = 0.749 − 2.8 and *P* = .271). Malawi College of Health Sciences students were more likely to adopt a surface approach to learning than students from Ekwendeni College of Health Sciences and there was statistical significance (OR = 3.716, and 95% CI = 1.912–7.225 and *P* = .001). The other demographic variables like gender and year of study showed some differences in the way students were adopting either surface approach to learning within the categories. However, the differences were not statistically significant. (See Table 2).

**Table 2:** Chi -square results for association of demographic characteristics of students and surface approach to learning

Variable	Surface approach (*N*) %	Not surface approach (*N*)%	X^2^	Df	*P* value
Age category (yrs)					
16–20	(31) 78	(9) 22	7.669	2	0.022
21–30	(107) 55	(88) 45			
31 and above	(11) 69	(5) 31			
Gender					
Male	(69) 62	(43) 38	0.442	1	0.516
Female	(80) 58	(59) 42			
Year of study					
One	(59) 60	(39) 40	3.007	2	0.22
Two	(46) 53	(41) 47			
Three	(44) 67	(22) 33			
College of study					
Ekwendeni	(38) 48	(41) 52	12.388	2	0.02
Nkhoma	(41) 54	(35) 46			
MCHS	(70) 73	(26) 27			

### Association of students’ demographic characteristics and deep approach to learning

The chi-square test for association was used to determine whether there was an association between students’ demographic characteristics and the adoption of a deep approach to learning. The results showed that there was no association between student age, gender, year of study, college of study, and adopting a deep approach to learning (p values = 0.815, 0.455, 0.441 and 0.086 respectively). See Table 3.

**Table 3:** Association of students’ demographic characteristics and deep approach to learning

Variable	Deep approach (*N*) %	Not deep approach (*N*)%	X^2^	df	*P* value
Age category (yrs)					
16–20	(33) 83	(7) 17	0.518	2	0.815
21–30	(168) 86	(27) 14			
31 and above	(14) 88	(2) 12			
Gender					
Male	(98) 87	(14) 13	0.559	1	0.455
Female	(117) 84	(22) 16			
Year of study					
One	(86) 88	(12) 12	1.777	2	0.441
Two	(71) 81	(16) 19			
Three	(58) 88	(8) 12			
College of study					
Ekwendeni			4.909	2	0.086
Nkhoma					
MCHS					

## Discussion

### Students’ approaches to learning among the participants

Most students had a deep approach to learning compared to a surface approach to learning.

Results were consistent with previous studies conducted in Malaysia, Pakistan, Geneva and Malawi [6, 9, 29, 30]. Chilemba found that most Malawian Undergraduate Bachelor of Science in nursing students adopted a deep approach to learning more than the surface approach to learning [9]. This shows that most Malawian nursing and midwifery students regardless of the level (diploma or degree level) of study adopt a deep approach to learning. This might mean that the nursing and midwifery colleges in Malawi offer a good learning environment for nursing and midwifery students. The Malawian Nursing and Midwifery education curriculums are competency based [25, 31]. This is also consistent with Bigg’s model and learning which says that several factors interact for teaching and learning to be effective [32]. Bigg’s 3P model of teaching and learning suggests that a good learning environment yields an adoption of a deep learning approach among students.

Similarly, Malaysian students adopted a deep approach (Mean = 2.685) more than a surface approach (Mean = 1.928) in a study which aimed at finding out the study process of public and private university students. In addition, a deep approach to learning was mostly used by registered nurse anesthetist (SRNA) education in Geneva [33]. A study in Malaysia also found a larger percentage of the undergraduate students in the faculty of medicine and health sciences at University of Putra in Malaysia had a deep approach to learning while only 21.3% had a surface approach to learning which showed that the majority of students had a deep approach to learning [34]. It was also found that a larger percentage of the undergraduate students in the faculty of medicine and health sciences at University of Putra in Malaysia had a deep approach to learning while only 21.3% had a surface approach to learning which showed that the majority of students had a deep approach to learning. Similarly, a comparative study in Pakistan found that medical students use more of deep approach than surface approach while students in general education used more of surface approach [19]. This could be attributed to the learner centered approach to learning that is practiced in nursing as well as medical education. The results are inconsistent with results from a study that was conducted to investigate the learning approaches of students in nursing education at the University of Hail in Saudi Arabia which showed that students were adopting both deep and surface approaches alike [35]. It is therefore important to ensure that a deep approach to learning should be encouraged as it has shown to impact students’ passing assessments, attaining competence and improve patient care in the long run [13, 14].

### Association of students’ demographic characteristics and their adopted approaches to learning

Younger age was associated with adopting a surface approach to learning. Students within the 16–20 years age group were mostly adopting the surface approach to learning than the older age categories. The results were inconsistent with a studies conducted in Singapore and in Nigeria which showed that age of student had no impact on the adoption of the surface approach to learning [17, 36]. However, it is important to note that students who are younger in most circumstances might not be very hardworking as they are in the stage that they are exploring more in their social life and as such would not pay much attention to what is being taught, instead they would just aim at passing thus adopting the surface approach more than their older mates. On the contrary a study Saudi Arabia discovered that there was a correlation between older age of students and both surface and deep approaches to learning [35].

College of study was associated with having a surface approach to learning. Malawi College of Health Sciences was more inclined to having a surface approach to learning than Nkhoma College of Nursing students as well as Ekwendeni College of Health Sciences and the difference was statistically significant (X^2^ = 12.388, *df* = 2, *P* = .002). Binary logistic regression results also showed that students from Malawi College of Health Sciences had higher odds of having a surface approach to learning than those from Nkhoma College of Nursing and Ekwendeni College of Health Sciences. This is consistent with previous findings which show that different learning environments affect students’ adoption of deep approach to learning. In another study that aimed at examining how students perceive learning activities, the acquisition of relevant knowledge, and educators’ enthusiasm/supportive attitudes impact on the students’ adoption of the deep approach to learning found that there was a positive relationship between students’ perceptions of learning activities and their use of an approach to learning [37]. These factors make up the learning environment which means that students who adopted a deep approach had a positive perception of their learning activities and felt that their nurse educators had supportive attitudes and vice versa.

It is also consistent with Bigg’s model of teaching and learning which says that there are presage, process and product factors. Presage factors are concerned with how the teaching and learning is designed at a particular college. The design is in terms of objectives set for the college, assessments planned for the students, plans on how lessons are to be delivered as well as the available college procedures and policies. These can differ from one college to another and can impact on adoption of deep approach to learning. Process factors are concerned with strategies used by teacher and students while learning is taking place [38]. This involves the approaches of teachers to teaching which could either be student- centered or teacher- centered in nature and these can differ from one nursing college to another which then impact on the students’ adoption of the deep approach to learning. Product factors are concerned with outcomes of the learning that has taken place which might be either rote, superficial learning showing that the student did not adopt a deep approach or deep understanding of the learnt content which shows that the student likely adopted a deep approach to learning.

Students’ gender and year of study showed no significance in relation to adoption of a surface approach to learning. This is in line with studies which were conducted in Singapore and Nigeria which found that there was no significance between students’ gender and sex and surface approach to learning [17, 36]. However, these findings are contrary to findings of studies which were conducted in Australia, Hongkong, Norway, Singapore, Malaysia and Saudi Arabia which showed that there was significance between students’ gender and their adopted approach to learning [11, 34]. There is no association between age of student, gender, year of study, college of study and adopting a deep approach to learning (p values = 0.815, 0.455, 0.441 and 0.086 respectively).

### Study limitation

The study was conducted in only three nursing schools out of the ten nursing schools that offer nursing and midwifery diplomas. Therefore, the results may not be generalizable to all nursing and midwifery technician students.

## Conclusion

Most students prefer a deep approach to learning than the surface approach to learning. However, the students use both deep and surface approach to learning. Students’ age and environment are the determining factors for approach to learning. Our study established that student in age range 16–20, and those from Malawi College of Health Sciences had high likelihood of adopting a deep approach to learning. This shows that having a younger age, and being at Malawi College of Health Sciences puts the student at a higher chance of adopting a surface approach to learning. More attention should be given to younger students during teaching and learning to promote deep learning. It is also important to ensure that nursing and midwifery educators create a student centered/ interactive learning environment to promote the adoption of a deep approach to learning in students which literature has shown that it improves student learning outcomes which later also improves patient care.

### Recommendation

More attention should be given to younger students during teaching and learning in order to promote a deep learning.

Future studies in the area of student approaches to learning should be done using a mixed methods approach so that the qualitative aspect of student approaches to learning concept should be captured.

More research should be done to find out why students from Malawi College of Sciences were adopting the surface approach to learning more than Nkhoma and Ekwendeni nursing colleges.

## Data Availability

The data used in this study are available from corresponding author (Dalitso Zolowere Chitokoto, dzolowere280@gmail.com) upon reasonable request.

## Abbreviations And acronyms

COM: College of Medicine
COMREC: College of Medicine Research and Ethics Committee
DA: Deep Approach
ECOHS: Ekwendeni College of Health Sciences
KCN: Kamuzu College of Nursing
KUHES: Kamuzu University of Health Sciences
MCHS: Malawi College of Health Sciences
R-SPQ-2F: Revised Two Factor Study Process Questionnaire
SA: Surface approach
SAL: Student Approaches to Learning
SPSS: Statistical Package for Social Science
